# Enhancing the flexibility of the peripheral regions within the catalytic cavity of a family 5 glycoside hydrolase CelB for improving catalytic efficiency

**DOI:** 10.3389/fbioe.2026.1931754

**Published:** 2026-10-26

**Authors:** Zihao Huang, Simeng Ding, Liangtao Xu, Jiahui Li, Lipeng Wang, Renjun Gao, Jun Zhou, Huaibin Shi

**Affiliations:** 1 State Key Laboratory of Analytical Chemistry for Life Science, School of Chemistry, Nanjing University, Nanjing, China; 2 China Tobacco Jiangsu Industrial Co., Ltd., Nanjing, China; 3 Key Laboratory for Molecular Enzymology and Engineering of Ministry of Education, School of Life Sciences, Jilin University, Changchun, China

**Keywords:** flexibility enhancement, GH5 endoglucanase, molecular dynamics simulation, peripheral regions, site-specific mutagenesis

## Abstract

Glycoside Hydrolase family 5 (GH5) endoglucanases are indispensable biocatalysts in lignocellulosic biorefining and their intrinsic catalytic performances play vital roles in process economics and enable broader industrial applicability. Herein, we engineered the catalytic domain of a bi-modular GH5 endoglucanase CelB from *Bacillus* sp. Strain N-4 by targeting non-conserved residues around peripheral, solvent-exposed regions. The site-specific mutagenesis identified two single-point mutants Y40G and T177N, which exhibited 1.64- and 1.55-fold increases in specific activity, respectively. Molecular dynamics (MD) simulations further revealed increased fluctuations in the corresponding peripheral regions, suggesting that this altered local backbone dynamics may contribute to the improved catalytic activity. Guided by these observations, iterative saturation mutagenesis was performed at Tyr40 and Thr177, focusing on small or hydrophilic residues to further explore the effects of substitutions at these peripheral, solvent-exposed regions. The final mutant Y40K/T177K exhibited enhanced catalytic efficiency, evidenced by *k*_*cat*_/*K*_*m*_ of 11.766 mL mg^-1^·s^-1^ and 2.719 mL mg^-1^·s^-1^ towards sodium carboxymethylcellulose and phosphoric acid-swollen cellulose, representing 2.73- and 1.78-fold enhancements over the wild-type enzyme, respectively. MD simulations further showed that Y40K/T177K exhibited enhanced flexibilities across multiple peripheral regions resulted in a more open catalytic cavity, thereby facilitating a greater displacement of cellohexaose ligand from the catalytic center. This behavior is consistent with an increased tendency toward glycan dissociation from the catalytic cavity, which may contribute to the enhanced catalytic turnover. Collectively, these findings underscore the functional importance of peripheral, solvent-exposed regions in modulating the catalytic performance of GH5 endoglucanases.

## 1 Introduction

Glycoside Hydrolase family 5 (GH5) is the largest and most diverse family of endoglucanases (EC 3.2.1.4), and holds historical significance as an earlier identified cellulase family (Henrissat et al., 1989; Suleiman et al., 2020). GH5 members are phylogenetically widespread and have been identified in prokaryotes, archaea, eukaryotes, and even certain viruses (Aspeborg et al., 2012; Ezeilo et al., 2017). According to the CAZy (Carbohydrate-Active enZYmes: http://www.cazy.org) database (Cantarel et al., 2009), GH5 enzymes exhibit functional diversity extending well beyond canonical endo-β-1,4-glucanase (cellulase) activity. For instance, certain GH5 enzymes may also function as endo-β-1,4-mannanase and endo-β-1,4-xylanase (Chen et al., 2012). Notably, p4818Cel5_2A, identified from the porcine gut microbiota can even catalyze the processive hydrolysis of cellulose into cellobiose and cellotriose (Wang et al., 2019). Such functional versatility renders GH5 endoglucanases promising candidates in diverse industrial sectors (Fan et al., 2021). Particularly, they serve as key enzymes in lignocellulosic biorefineries (Morozova et al., 2010; Zhang et al., 2011). Highly efficient and stable cellulases contribute to economical and sustainable industrial processes (Maleki et al., 2020). Accordingly, recent advances in cellulase engineering have mainly focused on improving hydrolysis efficiency and stress tolerance, aiming to develop low-cost and high-performance industrial enzymes (Contreras et al., 2020a).

To date, multiple structural engineering strategies have been established for the targeted modification of the catalytic domain of GH5 enzymes (GH5-CD). Zheng et al. focused on the loops exposed on the surface and optimized the residue at site 233 of *Gt*Cel5 from *Gloeophyllum trabeum* CBS 900.73, the variant N233G exhibited a 52% increase in the catalytic efficiency (*k*_*cat*_ */K*_*m*_). The Molecular Mechanics/Poisson Boltzmann Surface Area (MM/PBSA) calculations on the final 5-ns segments of molecular dynamics (MD) trajectories indicated that variant N233G exhibited a prominently lower binding free energy (−32.4 kcal mol^-1^) compared with the wild-type enzyme (−2.7 kcal mol^-1^), suggesting that enhanced flexibility of Loop 6 in N233G contributes to reinforce enzyme-substrate binding (Zheng et al., 2018). Wu et al. also investigated the dynamics of such loop regions in *Tf*Cel5A and found that Y361 W on loop 7 could facilitate product release, thereby increasing the activity by 40% (Wu et al., 2023). Lv et al. modified residues flanking the cellohexaose-binding site in, EG5C-1, a processive endoglucanase from *Bacillus subtilis*. By combining the alanine scanning and saturation mutagenesis, double mutant D70Q/S235 W showed a 2-fold increase in *k*_*cat*_ */K*_*m*_ for sodium carboxymethylcellulose (CMC-Na) hydrolysis. This improvement stemmed from new hydrogen bonds forming between the enzyme and cellohexaose, enhancing both substrate affinity and catalytic efficiency (Lv et al., 2021). The same group further engineered, EG5C-1 through the combination of consensus mutagenesis and loop engineering. The combinatorial mutant M3-1 (K91I/A198T/Q237D/V240 P) revealed 8.5- and 4.8-fold increases in catalytic efficiency towards CMC-Na and phosphoric acid swollen cellulose (PASC), respectively, which was attributed to the increased frequency of interactions between active site residues and substrate after mutagenesis (Lv et al., 2024). Zheng et al. engineered non-conserved residues on the outer surface of *An*Cel5A’s TIM-barrel fold using a consensus-guided stabilization strategy. Guided by ConSurf analysis, the native residues were replaced by the most frequent types and subsequently unstable mutants were filtered out via FoldX. The triple mutant N193A/T300P/D307 P showed 2-fold higher *k*_*cat*_ */K*_*m*_ than the wild-type towards CMC-Na (Zheng et al., 2022).

Although previous studies have probed the structure-function relationship of GH5-CDs, the roles of the peripheral, solvent-exposed regions surrounding catalytic cavity remain poorly understood. Herein, we engineered a bi-modular GH5 enzyme CelB from *Bacillus* sp. Strain N-4 by targeting non-conserved residues around peripheral, solvent-exposed regions. A structure- and sequence-guided engineering strategy was employed to enhance conformational flexibility. Based on this rationale, candidate sites revealed potentials for improving flexibility and specific activity were subjected to tailored, focused saturation mutagenesis restricted exclusively to small or hydrophilic substitutions (specifically those bearing polar or charged side chains) for further flexibility enhancement, thereby reducing the steric hindrance for catalytic dynamics. The advantageous mutants screened out from the library were biochemically characterized to evaluate their catalytic properties. MD simulations confirmed that the beneficial mutations enhanced flexibility across multiple peripheral regions thereby promoting catalytic cavity opening and increasing cellohexaose displacement from the catalytic center. These effects collectively contribute to improved catalytic turnover. The work provides a new avenue for design and engineering of GH5 enzymes, thus contributing to construct high-performance variants with application potential.

## 2 Materials and methods

### 2.1 Bacterial strains and materials

The encoding genes for cellulases were synthesized by Sangon Biotech Co., Ltd (Shanghai, China). The primers for site-specific or saturated mutation were synthe-sized by Comate Biosciences Co., Ltd. (Changchun, China). DL 10,000 and 15,000 DNA markers with QuickCut™ *Dpn* I were purchased from Takara Biomedical Technology Co., Ltd (Beijing, China). TransStart® FastPfu DNA Polymerase, *E. coli* Top10 and *E. coli* BL21 (DE3) competent cells were purchased from Beijing TransGen Biotech Co., Ltd (Beijing, China). Tricolor pre-stained protein marker (10–180 kDa) were from Sikejie Biotechnology Co., Ltd (Shandong, China). CMC-Na (DS = 0.7, 200–500 mPa s) was purchased from Shanghai Aladdin Biochemical Technology Co., Ltd (Shanghai, Chi-na). Avicel was purchased from Macklin Biochemical Co., Ltd. (Shanghai, China). The SYPRO^TM^ Orange protein gel stain (5,000× concentrate in DMSO) was purchased from Invitrogen (Thermo Fisher Scientific Inc., Waltham, MA, USA).

### 2.2 Gene synthesis, expression and activity detection of CelB and other candidates

Given that CelB with other alternative enzymes (Bl0147 and CelN) are naturally secreted by their native host strains, their N-terminal signal peptides were predicted and removed using SignalP-5.0 (https://services.healthtech.dtu.dk/services/SignalP-5.0/) (Almagro Armenteros et al., 2019). Residue numbering throughout this study was based on the mature protein sequence after removal of the predicted signal peptides. The remaining coding sequences were codon-optimized for expression in *E. coli*, synthesized, and subcloned into pET21a (+) between the *Bam*H I and *Xho* I restriction sites. Recombinant plasmids were transformed into *E. coli* BL21 (DE3). Positive clones were cultured in Luria-Bertani (LB) medium with kanamycin sulfate (50 μg/mL) at 37 °C, 160 rpm until OD_600_ reached 0.8. Protein expression was induced with 0.2 mM isopropyl β-D-1-thiogalactopyranoside (IPTG) at 25 °C. Cells were harvested by centrifugation, resuspended in 50 mM phosphate buffer (pH 7.0), and lysed via pulsed sonication (200 Hz, 6 min). Crude enzymes (supernatants) were obtained by centrifugation (12,000 rpm, 10 min) to remove debris. Expression levels were assessed by SDS-PAGE. Target protein band intensities were quantified using ImageJ (Schneider et al., 2012). The total soluble protein concentrations of the crude enzyme extracts were determined via BCA assay. Enzymatic activities were measured using appropriately diluted crude enzymes. Each reaction mixture contained 100 μL diluted enzyme and 100 μL CMC-Na (DS = 0.7, 200–500 mPa s, 10 mg/mL). After incubation at 30 °C for 5 min, reactions were terminated by adding 600 μL DNS reagent and boiled for 5 min. Absorbance was detected under 540 nm and the standard curve fitting was performed using glucose. One unit (U) of activity was de-fined as the amount of enzyme releasing 1 μmol reducing sugar per minute. Final specific activities were calculated using Equation 1 below:

$$\text{Activitiy }\left(U/\text{mg}\right)=\frac{c_{\text{sugar}}\times 0.2\times n}{t\;\left(\min\right)\times \left(c_{\text{total}}\times p_{\text{target}}\times 0.1\right)}$$ (1)

where c_sugar_ (mM) represents the concentrations of reducing sugar calculated from standard curve, n represents the dilution factor, c_total_ (mg/mL) and p_target_ represent the total soluble protein concentration and proportion of target enzyme respectively.

### 2.3 Single point mutagenesis of non-conserved residues within peripheral regions

Molecular docking was first performed to identify potential hotspots involved in substrate recognition by CelB. The three-dimensional structure of CelB was obtained from the AlphaFold database (AF-P06565-F1) (Varadi et al., 2022). The cellohexaose ligand was extracted from the reported structure of a cellobiohydrolase I from *Trichoderma reesei* (PDB ID:7CEL) (Divne et al., 1998). Prior to molecular docking, the receptor (CelB) was protonated and subjected to energy minimization, whereas the ligand (cellohexaose) underwent energy minimization. Docking simulations were performed using the CDOCKER algorithm implemented in Discovery Studio 2021. The ligand binding site was defined as a spherical region encompassing the functionally implicated residues (His35, Trp39, Tyr40, Tyr40, His101, Glu139, Tyr176, Glu228, Ala234, Thr235, W262, Lys267, Asp268, and Glu269) annotated in UniprotKB database (Accession: P06565). The final docking sphere was centered at X: 1.638, Y: 7.981, Z: 11.852, with a radius of 15.198 Å. The CDOCKER parameters “Top Hits” and “Pose Cluster Radius” were set to 10 and 0.5 Å, respectively to produce more possible poses. Residues located within a 5 Å radius of the cellohexaose molecule were initially selected as candidate mutagenesis sites. Subsequently, evolutionary conservation analysis was carried out using the ConSurf server (https://consurf.tau.ac.il/consurf_index.php) (Ashkenazy et al., 2016) to filter out highly conserved residues that were likely to be structurally or functionally indispensable, thereby facilitating the rational selection of mutagenesis targets based on residue variability. Finally, Trp39, Tyr40, Thr177, Leu210, and Ser265 were chosen for further mutagenesis. The relative solvent-accessible surface area (RSA) analysis and corresponding structural diagrams were all accomplished in Open-Source PyMoL 2.4.0a0.

The single-point mutations were introduced by whole-plasmid mutagenic PCR using CelB-pET21a (+) as template. Mutagenic primers were listed in Supplementary Table S1. After *Dpn* I digestion to selectively degrade the methylated template, the amplified products were ligated by T4 ligase and transformed into *E. coli* BL21 (DE3). Recombinant protein expression was carried out as described in Section 2.3. To facilitate the identification of advantageous mutations, all single mutants were purified by Ni^2+^-NTA affinity chromatography, and then subjected to the activity detection with a protein concentration of 0.05 mg/mL.

### 2.4 Iterative, focused saturated mutagenesis of Tyr40 and Thr177

Focused saturated mutagenesis was first performed at position Tyr40 using two complementary strategies: (i) substitutions with selected small residues were achieved via standard site-specific mutagenesis using the primers listed in Supplementary Table S2; and (ii) substitution with hydrophilic residues (Lys, Asn, Gln, His, Glu, and Asp) were introduced using degenerate primers with codon “^5’^-VAW-^3’^” listed in Supplementary Table S4. Subsequently, using Y40K as the template, the iterative saturation mutagenesis at Thr177 was carried out following the same procedure with the primers listed in Supplementary Tables S2, S3. To increase library diversity, PCR reactions for constructing the focused library with hydrophilic substitutions were performed in duplicate for each target site. Individual transformants were picked and cultured in LB medium for crude enzyme preparation and initial activity screening. Colonies exhibiting higher enzymatic activity than the corresponding control strain were subjected to plasmid sequencing before purification by Ni^2+^-NTA affinity chromatography for subsequent activity assays.

### 2.5 MD simulations

The MD simulations for CelB and variants were carried out using GROMACS 2025.2. The site-specific mutations were introduced using the Mutagenesis Wizard plugin in PyMOL and then minimized in Discovery Studio 2021. The protein parameterized with the AMBER14SB force field and solvated with the SPC/E water model (Meyer et al., 2019). Each system was placed at the center of a cubic simulation box with a minimum distance of 1.2 nm between the protein and the box boundaries. The systems were neutralized by replacing solvent molecules with sodium ions. Energy minimization was conducted using 50,000 steps of steepest descent followed by the conjugate gradient energy minimization, with a F_max_ tolerance of 1,000 kJ/mol/nm. Following energy minimization, the system was equilibrated under NVT conditions with position restraints for 100 ps, followed by an additional 100 ps under NPT conditions. A 100-ns production simulation was then carried out using a 2-fs time step. Structural coordinates were saved at 10-ps intervals for Root Mean Square Deviation (RMSD) analysis. Root Mean Square Fluctuation (RMSF) values were calculated from the equilibrated trajectories (60–100 ns). The complete RMSD curves for CelB and variants were summarized in Supplementary Figure S8. To assess whether enhanced flexibility in peripheral regions facilitated glycan chain expulsion and enzyme dissociation, MD simulations were further performed on the enzyme-cellohexaose complexes of CelB and Y40K/T177K. The force field parameters for cellohexaose were generated using Sobtop (Tian Lu, Version 2026.1.16; http://sobereva.com/soft/Sobtop, accessed May 2026). Simulation protocols were basically same to the aforementioned. Subsequent analyses were conducted over the 100-ns trajectories. To monitor the changes in openness of catalytic cavity (defined based on the hotspots within 5 Å proximity of the cellohexaose ligand), solvent accessible surface area (SASA) analysis of catalytic cavity was performed using the probe radius of 0.14 nm. SASA values were calculated at 100-ps intervals and subsequently averaged across the trajectory. Furthermore, the dynamic changes of distance between the center of mass (COM) of the catalytic residues (Glu139 and Glu228) and the cellohexaose ligand were monitored and analyzed. The COMs of catalytic residues and cellohexaose ligand in the structure model were calculated using the “centerofmass” command in Open-Source PyMoL 2.4.0a0.

### 2.6 Biochemical characterization (temperature/pH profiling, thermostability, impact of metal ion, Michaelis-Menten kinetic analysis)

To assess the functional impact after mutagenesis, the temperature and pH profiles of CelB and mutants were detected. For temperature profiles, enzymatic activity was measured over a temperature range of 20 °C–90 °C by mixing 100 μL purified enzyme solution (0.05 mg/mL) with 100 μL CMC-Na (DS = 0.7, 200–500 mPa s, 10 mg/mL). Reaction conditions and the DNS assays were as described above. For pH profiling, enzyme activity was determined using 50 mM citrate-Na_2_HPO_4_ (pH 4.0–7.0) and phosphate buffer (pH 7.0–8.5). The effects of metal ions were evaluated after incubating 0.05 mg/mL enzymes with 10 mM different metal ions (Mg^2+^, Ca^2+^, Co^2+^, Mn^2+^, Zn^2+^, Cu^2+^, Fe^2+^, Fe^3+^, K^+^, Na^+^) at room temperature for 5 min, followed by activity measurement. All experiments were performed in triplicate.

For thermostability analysis, thermal inactivation assays were conducted at 30 °C and 60 °C in a constant-temperature water bath. Residual activities were measured at regular time intervals to evaluate whether enhanced flexibility in the peripheral regions adversely affected enzyme thermostability. Subsequently, thermodynamic conformational stability was assessed by differential scanning fluorimetry (DSF) using a real-time PCR instrument. Protein samples (3 μM) in 50 mM, pH 7.0 PB buffer were mixed with SYPRO^TM^ Orange dye at a final concentration of 5×, heated from 20 °C to 99.9 °C at a rate of 0.05 °C/s, and fluorescence was monitored (excitation at 470 nm, emission at 570 nm). The melting temperature (*T*_m_) was determined from the derivative (dF/dT) of the fluorescence melting curve.

Kinetic parameters were determined against CMC-Na (DS = 0.7, 200–500 mPa s, 10 mg/mL) and PASC as substrates. Enzyme assays were performed at 50 °C and pH 6.5 with substrate gradients ranging from 2.5 to 20.0 mg/mL. PASC was prepared by swelling 2.0 g Avicel with 50 mL 85.0% (w/v) phosphoric acid on ice for 1 h, diluting with 0.8 L distill water and stirring for 1 h. After vacuum-filtering, the precipitate was washed with 2.0 L distilled water, and then neutralized by adding 1.5 L, 1.0% (w/v) NaHCO_3_. Finally, thoroughly rinsed with distilled water. Moreover, owing to the intrinsic insolubility of PASC in aqueous media, enzymatic reactions with PASC were carried out in a temperature-controlled shaker set at 200 rpm. To ensure homogeneous substrate dispersion and prevent sedimentation of PASC particles, the reaction tubes were positioned horizontally, thereby maintaining suspension uniformity during incubation period. After data collection, Michaelis-Menten constant (*K*_*m*_), turnover number (*k*_cat_), and apparent second-order rate constant (*k*_cat_ */K*_*m*_) were calculated, respectively, using allosteric sigmoidal in GraphPad Prism 7.

### 2.7 Statistical analysis

Statistical significance of changes in enzymatic activity before and after site-directed mutagenesis was determined using two-tailed unpaired t-tests implemented in GraphPad Prism 7. This statistical approach was applied consistently across all rounds of mutagenesis to identify mutants with statistically significant enhancements in catalytic activity. Significance levels were defined as follows: *p < 0.05, **p < 0.01, ***p < 0.001, and ****p < 0.0001.

## 3 Results

### 3.1 Identification, sequence and docking analysis of CelB

Recently, we identified a bi-modular GH5 endoglucanase, CelB (GenBank: AAA22299.1), through phylogenetic analysis (Supplementary Figure S1A) using the canonical bi-modular GH5 endoglucanase *Bs*Egls (UniProt ID: A5A671, 499 aa) as a reference (Aa et al., 1994; Santos et al., 2012). As summarized in Table 1, prior studies have predominantly centered on single-domain GH5 endoglucanases. In contrast, CelB features an underexplored structural paradigm (Supplementary Figure S1B), featuring a canonical GH5 catalytic domain (D1–S309), a distinctive chitin-binding domain (ChtBD3, G338–N383), and a proline-riched linker (PR-linker, D310–P337) that mediates interdomain connectivity. This modular architecture constitutes a functionally informative model for advancing structural and functional understanding of the GH5 enzyme family. Among the three phylogenetically selected candidates (BL0147, CelB and CelN), CelB exhibited robust soluble expression (Supplementary Figure S1C), with approximately 40% of the total expressed protein in supernatant. Its specific activity towards CMC-Na was 2.813 U/mg. Further sequence and structural alignments showed that CelB’s GH5-CD shared 81.61% sequence identity and near-identical fold (RMSD of 0.157 Å) with, EG5C-1, a new, processive endoglucanase previously characterized in Lv’s work (Lv et al., 2021). The combination of a conserved catalytic core and a unique modular architecture suggested that CelB represented a promising scaffold for structure-guided engineering. Accordingly, we undertook a systematic structure-function investigation of CelB and proposed an effective engineering strategy to further enhance its catalytic performance.

**TABLE 1:** Summary of representative studies on GH5 endoglucanases along with a comparative analysis of the structural architectures of their target enzymes.

Enzymes	Sources	Structure paradigm	Relevant study
Cel5A	*Thermoascus aurantiacus*	Single-domain GH5-CD	(Leggio and Larsen, 2002)
Cel5A	Soil metagenome	Single-domain GH5-CD	(Voget et al., 2006)
CelDZ1	Icelandic Hot spring Isolate	Single-domain GH5-CD	(Zarafeta et al., 2016)
Cel5R	Soil metagenome	Single-domain GH5-CD	(Garg et al., 2016)
*Fm*EG	*Fomitiporia mediterranea* MF3/22	Single-domain GH5-CD	(Pan et al., 2016)
*Gt*Cel5	*Gloeophyllum trabeum* CBS 900.73	Single-domain GH5-CD	(Zheng et al., 2018)
Cel5E	*Clostridium thermocellum*	Single-domain GH5-CD	(Torktaz et al., 2018)
GH5CelA/B	Termite microbiome	Single-domain GH5-CD	(Guerrero et al., 2020)
*Pv*Cel5A	*Penicillium verruculosum*	Single-domain GH5-CD	(Contreras et al., 2020b)
Cel5A	*Martelella endophytica* strain YC6887	Single-domain GH5-CD	(Khan et al., 2021)
EG5C-1	*Bacillus subtilis* BS-5	Single-domain GH5-CD	(Lv et al., 2021)
*An*Cel5A	*Aspergillus niger*	Single-domain GH5-CD	(Zheng et al., 2022)
*Tf*Cel5A	*Thermobifida fusca*	Single-domain GH5-CD	(Wu et al., 2023)
*Bs*Cel5A	*Bacillus subtilis* 168	Bi-modular GH5-CD + *CBM3 (family 3 carbohydrate-binding module)*	(Santos et al., 2012)
CEL-5A	*Bacillus subtilis* 1AJ3	Bi-modular GH5-CD + CBM3	(Ma et al., 2020)
*Mt*Glu5	*Meiothermus taiwanensis* WR-220	Bi-modular GH5-CD + inserted CBM29 like domain	(Ye et al., 2022)
*Bs*EGL	*Bacillus subtilis* DLG	Bi-modular GH5-CD + CBM3	(Meng et al., 2026)
CelB	*Bacillus* sp. Strain N-4	Bi-modular GH5-CD + ChtBD3	This study

Molecular docking (Figure 1) was first employed to identify promising regions and residues potentially involved in catalytic process. Based on a 5 Å proximity criterion centered on the cellohexaose ligand, the following residues were identified as hotspots potentially involved in catalytic dynamics: His35, Trp39, Tyr40, Tyr66, His101, Leu103, Asn138, Glu139, Thr177, Trp178, Gln180, His200, Thr205, His206, Leu210, Glu228, Ala234, Gly236, Trp262, Thr265, Lys267, Glu269. However, Consurf analysis (Supplementary Figure S2) eliminated most of these residues from further consideration, as they exhibited conservation scores greater than 8, indicating likely functional or structural significance. The excluded residues included His35, Tyr66, His101, Leu103, Asn138, Glu139 (acid/base catalyst), Trp178, Gln180, His200, Thr205, His206, Glu228 (nucleophile), Ala234, Gly236, Trp262, Lys267, and Glu269.

**FIGURE 1:**
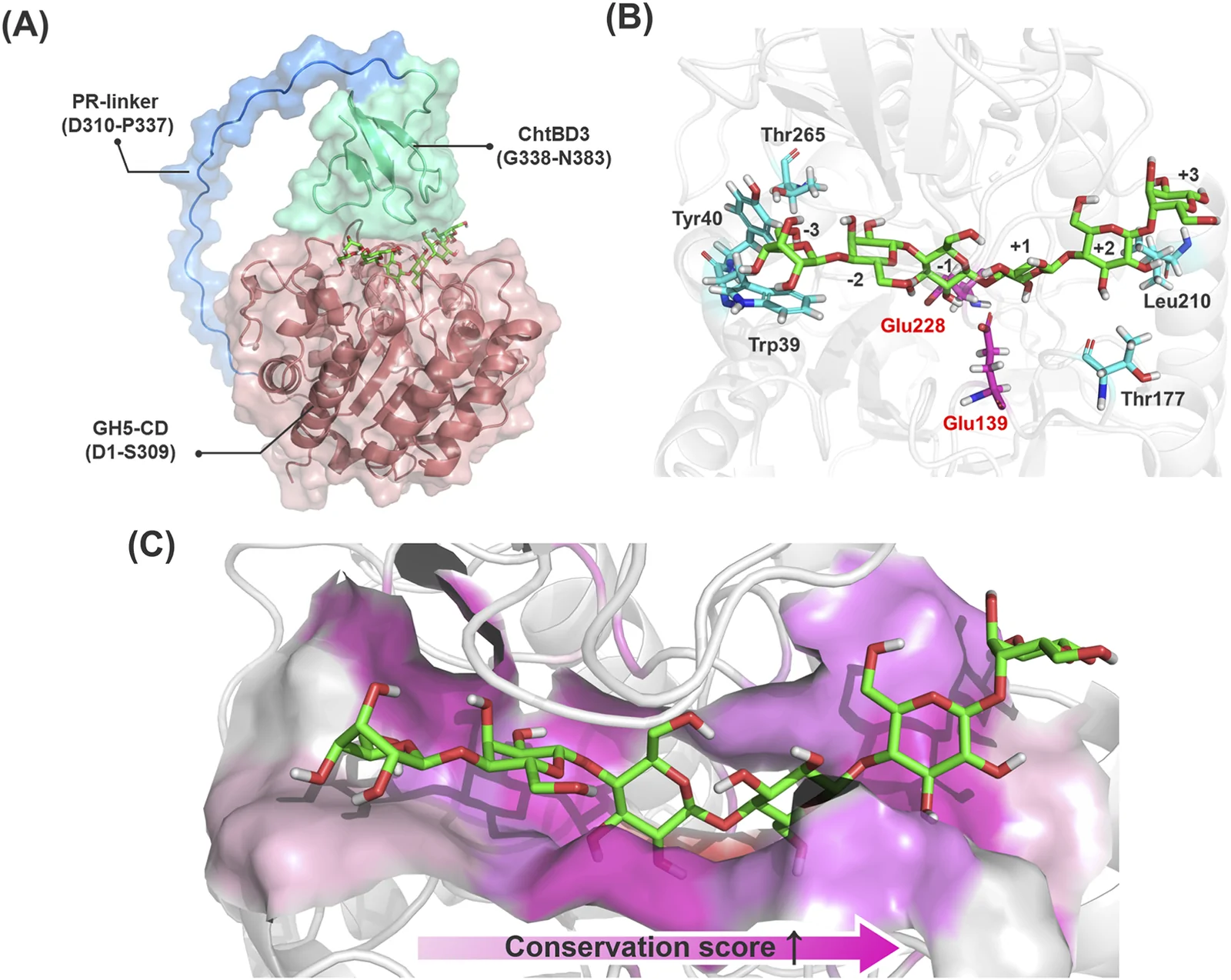
Selection for mutation sites based on the structural analysis of CelB-cellohexaose complex. **(A)** Overview for the complex generated from docking. **(B)** Non-conserved sites reside around peripheral, solvent-exposed regions were selected for subsequent mutagenesis. **(C)** Conservation mapping for catalytic cavity of CelB.

As shown in Figure 1B, the remaining 5 sites (Trp39, Tyr40, Thr177, Leu210 and Thr265) were located within 5 Å of the docked cellohexaose ligand but were relatively distant from the catalytic center (Glu139 and Glu228). These residues were positioned in peripheral regions near the solvent-exposed surface of the catalytic domain, suggesting potential roles in modulating the entry and expulsion of glycan rather than direct participation in catalysis. Conservation mapping (Figure 1C) revealed a clear spatial gradient, with residue conservation generally increasing toward the catalytic center and particularly high conservation observed around Glu139 and Glu228. Consequently, this highly conserved region is hard to tolerate substitutions and represents a low-priority target for rational engineering relative to the peripheral regions discussed above. A recent study on human kynureninase demonstrated that targeting mutagenesis to flexible, high-B-factor residues at the enzyme periphery produced a variant with markedly enhanced catalytic activity, revealing that remote flexibility modulation can influence catalysis (Karamitros et al., 2022). This was similarly demonstrated in RNase A, where the flexibility of peripheral loop1 played a pivotal role in the rate-limiting product release step. Restricting the mobility of loop1 led to a 10-fold decrease in release rate (Doucet et al., 2009). Likewise, structure-guided engineering of D-carbamoylase *Ni*HyuC showed that enhancing the flexibility of a peripheral, solvent-exposed loop (residues 200–207) improved substrate access, thereby significantly boosting catalytic efficiency (Liu et al., 2020). The above research indicates the peripheral regions of catalytic domain are not merely passive conduits but dynamic determinants of catalysis. Engineering of these peripheral regions holds demonstrable potential to improve catalytic performance.

Structural analysis (Figure 1B) showed that Trp39, Tyr40, and Thr265 were located near the −3 subsite, whereas Thr177 and Leu210 oriented toward the +2 subsite. All five residues resided around the interface between the catalytic cavity and the solvent environment, supporting their potential involvement in the accessing and expulsion of glycan chains. Uniquely, Thr265 was fully buried beneath bulky Trp39, Tyr40, and Phe43. Mutation at this position could therefore perturb the local packing arrangement and potentially influence the conformation of the surrounding residue cluster. Relative solvent-accessible surface area (RSA) analysis (Supplementary Figure S3) yielded consistent results. Trp39 and Thr177 were fully exposed, with RSA values exceeding 50%; Tyr40 and Leu210 were partially exposed, with RSA values falling between 20% and 50%; and Thr265, consistent with its buried structural position, was completely inaccessible to solvent (RSA = 0%) (Fraczkiewicz and Braun, 1998). Accordingly, the five candidate residues were initially substituted guided by Consurf-derived evolutionary profiling (Supplementary Figure S3). Mutation design prioritized substitutions that recur at higher frequency in the evolutionary profile. To alleviate local steric constraints and enhance conformational flexibility, volume-reducing substitutions were introduced first, including W39F, Y40G, Y40I, L210V, and T265 S. Furthermore, for solvent-exposed sites Trp39 and Thr177, substitutions were then selected on the basis of protein surface hydration, a recognized determinant of mobility and conformational dynamics in aqueous environments (Zhang et al., 2007; Bone, 2008; Qin et al., 2016). Based on the premise that hydration of solvent-exposed sites can influence local conformational mobility in aqueous solution, W39 T replaced the bulky aromatic indole side chain of Trp with the smaller hydroxyl-containing side chain of Thr, thereby reducing side-chain volume and aromaticity while introducing a potential hydrogen-bonding partner for solvent water. Likewise, T177 N substituted Thr with Asn to increase hydrophilicity, potentially strengthening interactions with the surrounding solvent and improving local hydration. Together, this structure-guided, evolution-informed approach provided a rational basis for tuning the flexibility of peripheral regions, which in turn might modulate the glycan access and expulsion, ultimately affecting catalytic performance.

### 3.2 Single-point mutagenesis

Site-directed mutagenesis was performed via whole-plasmid PCR to introduce the aforesaid substitutions. As shown in Figure 2A, the linear PCR products produced the expected band at approximately 6,558 bp. Following transformation into *E. coli* BL21 (DE3), sequencing verification and expression screening, the correct strains were subjected to mass-cultivation and purified using Ni^2+^-NTA affinity chromatography. SDS-PAGE analysis confirmed that all purified mutants exhibited ≥90% purity (Figure 2B). Notably, the W39 T mutant failed to realize soluble expression, whereas W39 F successfully expressed (Supplementary Figure S4). Structural analysis revealed that the native, aromatic Trp39 formed a Pi-Pi T-shaped interaction with the highly conserved Tyr66. Therefore, the aromatic side chain at position 39 appears to be important for maintaining local structural integrity and soluble expression. Endoglucanase activity assays using CMC-Na as substrate (Figure 2C) further indicated that W39F, Y40G, Y40I and T177 N showed varying degrees of activity enhancements. Among these single-point mutants, Y40G (4.622 U/mg) and T177 N (4.357 U/mg) revealed prominent enhancements in specific activity which increased by 1.64-fold and 1.55-fold respectively. RMSF analysis (Figure 2D) further demonstrate that Y40G slightly increased the local fluctuations within Ser33-Gly41. This enhancement is consistent with the greater backbone conformational freedom generally associated with glycine, which lacks a Cβ atom and imposes relatively minimal steric constraints (Yan and Sun, 1997; Jacob et al., 1999; Charoenwongpaiboon et al., 2024). Similarly, T177 N also showed elevate fluctuations within Ile172-Asp181, likely attributable to the higher hydrophilicity of Asn relative to Thr (Wimley and White, 1996), thereby promoting side-chain hydration and mobility in aqueous environment. Collectively, the substitutions at site 40 and 177 showed tendencies to enhance the flexibility in their respective peripheral regions. Therefore, they were prioritized for site-saturation mutagenesis to systematically explore optimal substitutions capable of further flexibility change.

**FIGURE 2:**
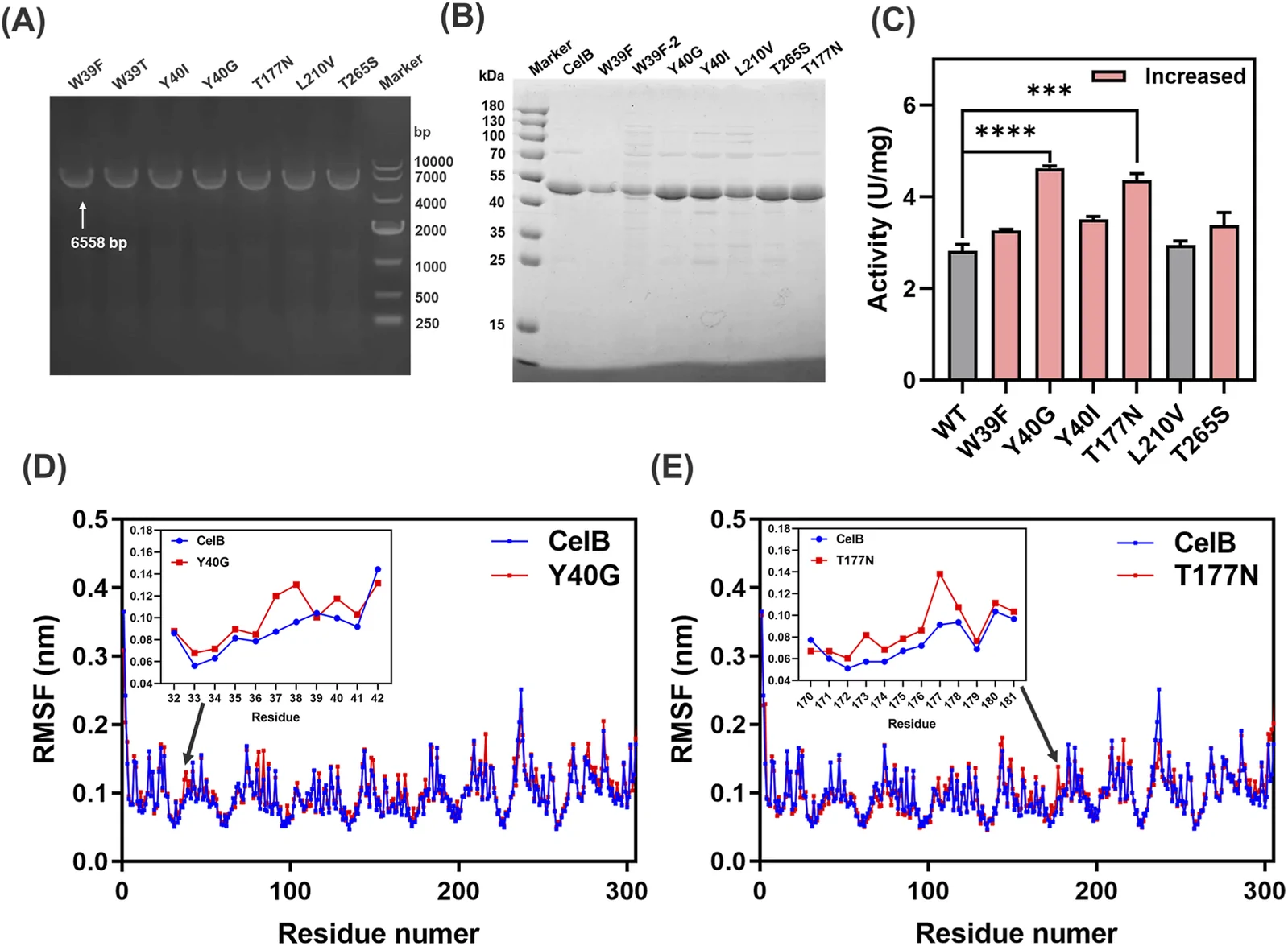
Screening of single-point mutants (W39F, W39T, Y40G, Y40I, T177N, L210V, and T265 S). **(A)** Whole-plasmid mutagenetic PCR result to introduce site-specific mutation. **(B)** Purification for single-point mutants. **(C)** Specific activities of single-point mutants towards CMC-Na (statistical significances were analyzed between Y40G, T177 N and WT, ***p < 0.001, ****p < 0.0001). **(D)** RMSF comparison between Y40G and WT to evaluate the flexibility change induced by mutation. **(E)** RMSF comparison between T177 N and WT.

### 3.3 Focused saturated mutagenesis of Tyr40

Tyr40 was selected for the first-round saturated mutagenesis owing to its better activity enhancement. To comprehensively evaluate the potential of Tyr40 substitution to enhance conformational flexibility, we considered not only smaller residues but also hydrophilic ones. Although evolutionary analysis using ConSurf (Supplementary Figure S3) revealed that hydrophilic residues like Gln (3%), Lys (1%), and Glu (1%) rarely occur at this position, the solvent-exposed location of Tyr40 and the experimentally improved activity achieved with the Y40G mutation led us to reason that local flexibility around this site may be contribute to catalytic performance. We therefore hypothesized that introducing either small or hydrophilic residues at position 40 could further increase conformational flexibility in the aqueous environment, with potential consequences for catalytic activity. To test this, a focused mutagenesis library was designed to incorporate small (Ala, Val, Ser) and representative hydrophilic residues with polar or charged side-chains for better hydration. Substitutions of small residues were introduced by site-specific primers while the hydrophilic library was constructed via a degenerate codon “^5’^-VAW-^3’^” incorporated into the same primer set (Supplementary Table S3). It encoded six hydrophilic residues (Lys, Asn, Gln, His, Glu and Asp). Meanwhile, each residue appeared only once in this library to reduce the library volume and increase the hit rate. Notably, arginine was deliberately excluded because its bulky guanidine group could introduce redundant interactions which was highly probable to create passive impact on original interaction networks. Previous studies have demonstrated that guanidinium group of Arg is prone to engage in non-specific cation–Pi and electrostatic interactions (Kumar and Nussinov, 2002; Kumar et al., 2018). Additionally, arginine always remains positively charged within the protein interior (fully protonated when pH ≤ 10) (Harms et al., 2011). Introducing arginine is likely to reduce the thermodynamic stability of protein. Moreover, introducing codons of Arg (e.g., ^5’^-CGT-^3’^) would enrich library capacity from 6 to 12 which increase the screening workload. Taken all together, Arg was excluded from this library.

Based on this methodology, site-specific mutations were introduced via whole-plasmid mutagenic PCR, and the resulting linear products exhibited the expected lengths (Supplementary Figure S5A). To minimize the impacts of cultivation and induction conditions, the enzymatic activities of mutated colonies were detected and compared to the CelB cultivated and induced in the same batches. As shown in Figure 3A, several crude enzymes prepared from this focused library revealed elevated activities in the initial screening, indicating that Tyr40 was a promising site that tolerated multiple substitutions. The variants exhibiting relative activities exceeding 120% were subjected to plasmid extraction and sequencing. After screening, the mutants carrying small residue substitutions and those identified from focused library were all purified by Ni^2+^-NTA affinity chromatography before final activity confirmation (Figure 3B). The specific activities of final resulting mutants were presented in Figure 3C. Among the variants identified from this focused library, the two single-point mutants Y40K (6.896 U/mg) and Y40Q (5.390 U/mg) exhibited the most pronounced activity improvements, which were 2.58-fold and 2.02-fold higher than that of the wild-type, respectively. Both variants outperformed the previously identified top-performing variant Y40G (4.622 U/mg) as well as other mutants bearing substitutions with smaller residues. Given Lys’s well-documented hydrophilicity (Kyte and Doolittle, 1982), increasing the hydrophilicity of the residue at position 40 was more beneficial than simply reducing side-chain size. RMSF analysis showed that Y40K exhibited increased backbone flexibility in His35-Asn45, as well as in several additional regions, including Ala64-Val78 and Ser263-Ser290 (Figure 3D). Notably, these regions are located predominantly at the periphery of the catalytic cavity and are solvent-exposed. Beyond the effects arising from enhanced hydrophilicity of Y40K, diminished aromaticity also contributes to the flexibility change of corresponding regions. As shown in the interaction visualization of Tyr40 (Figure 3F), its bulky, aromatic sidechain originally engages in hydrophobic packing with His35 and Lys267. Substitution with Lys disrupted these interactions, providing a plausible structural basis for the increased local and distal fluctuations observed in Y40K. For instance, the perturb of inter-regional interactions like Pi-Alkyl interactions between Tyr40 and Lys267 contribute to the flexibility enhancement of Ser260-Ser290. Similarly, the increased fluctuations observed in His101-Lys112 may be associated with its structural coupling to Ala64-Val78 through several noncovalent interactions (e.g., alkyl-Pi interaction between Ile73 and Tyr111; hydrogen bonding between Thr67 and His101). Collectively, the Y40K substitution induces widespread flexibility enhancement through a coordinated network of local and long-range noncovalent interactions. A similar phenomenon has been demonstrated by Tiwari’s research which found that long-range dynamic correlations could propagate across multiple β-α units within the TIM-barrel scaffold (Tiwari and Reuter, 2016). Therefore, the enhanced flexibility observed in His35-Val46 (connecting the β1-α1 unit of CelB) may exert a more extensive impact.

**FIGURE 3:**
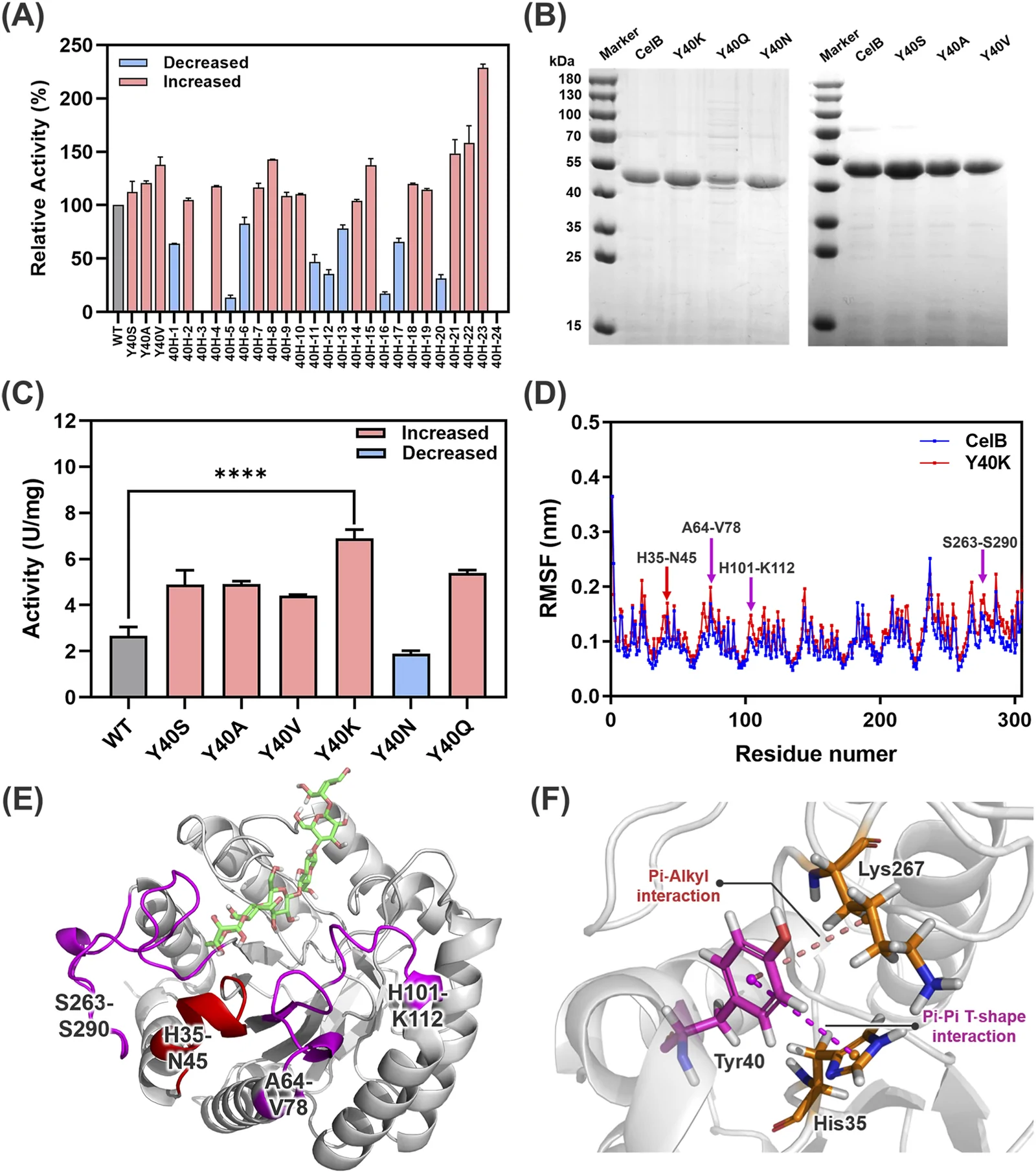
Focused, saturated mutagenesis of Tyr40 with representative small residues or hydrophilic ones. **(A)** Initial screening for focused library of Tyr40. **(B)** Purification for the mutants screened out from the library. **(C)** Specific activities of purified mutants towards CMC-Na (statistical significances were analyzed between the best Y40K and WT, ****p < 0.0001). **(D)** RMSF comparison between Y40K and WT. **(E)** Y40K induced the flexibility enhancement of its local region (H35-V46, colored in red) and adjacent regions (colored in purple). The pose of cellohexaose was shown to corroborate the spatial relationship between the ligand and these regions. **(F)** The hydrophobic packing interaction between Tyr40 and its neighboring His35 and Lys267 analyzed using Discovery Studio 2021.

### 3.4 Iterative, focused saturated mutagenesis of Thr177 on the basis of Y40K

The iterative, focused saturated mutagenesis of Thr177 was performed using Y40K as the template, targeting substitutions with small or hydrophilic residues. The resulting linear products exhibited the expected lengths (Supplementary Figure S5B). Initial screening of the Thr177-focused library (Figure 4A) revealed that most variants exhibited enhanced catalytic activity than the Y40K parental strain, which was cultivated and induced under identical experimental conditions. Notably, the mutants in hydrophilic library generally showed better activities when compared with those bearing small-residue substitutions (Y40K/T177 A or G or S). To assess expression behavior, a subset of variants from this focused library, including three representative small-residue variants, were selected for expression detection (Supplementary Figure S6). Critically, substitution with Ala, Gly, or Ser did not significantly impair expression levels, indicating that the relatively lower activities observed for these three mutants were attributable to intrinsic functional deficits rather than insufficient protein yield. Based on these results, variants carrying hydrophilic substitutions performed better and were therefore selected for sequence verification and further characterization. As shown in Figure 4C, the best double mutant Y40K/T177K gained further activity enhancement to 9.107 U/mg based on Y40K which was 3.2-fold higher relative to the wildtype. RMSF analysis (Figure 4D) indicates that, in addition to the regions displaying increased flexibility in Y40K, the incorporation of the T177K substitution further increases the flexibility of Gly174-Asp181 (Figure 4E). Collectively, Y40K/T177K displays elevated conformational flexibility across multiple peripheral regions that enclosing the cellohexaose-binding site.

**FIGURE 4:**
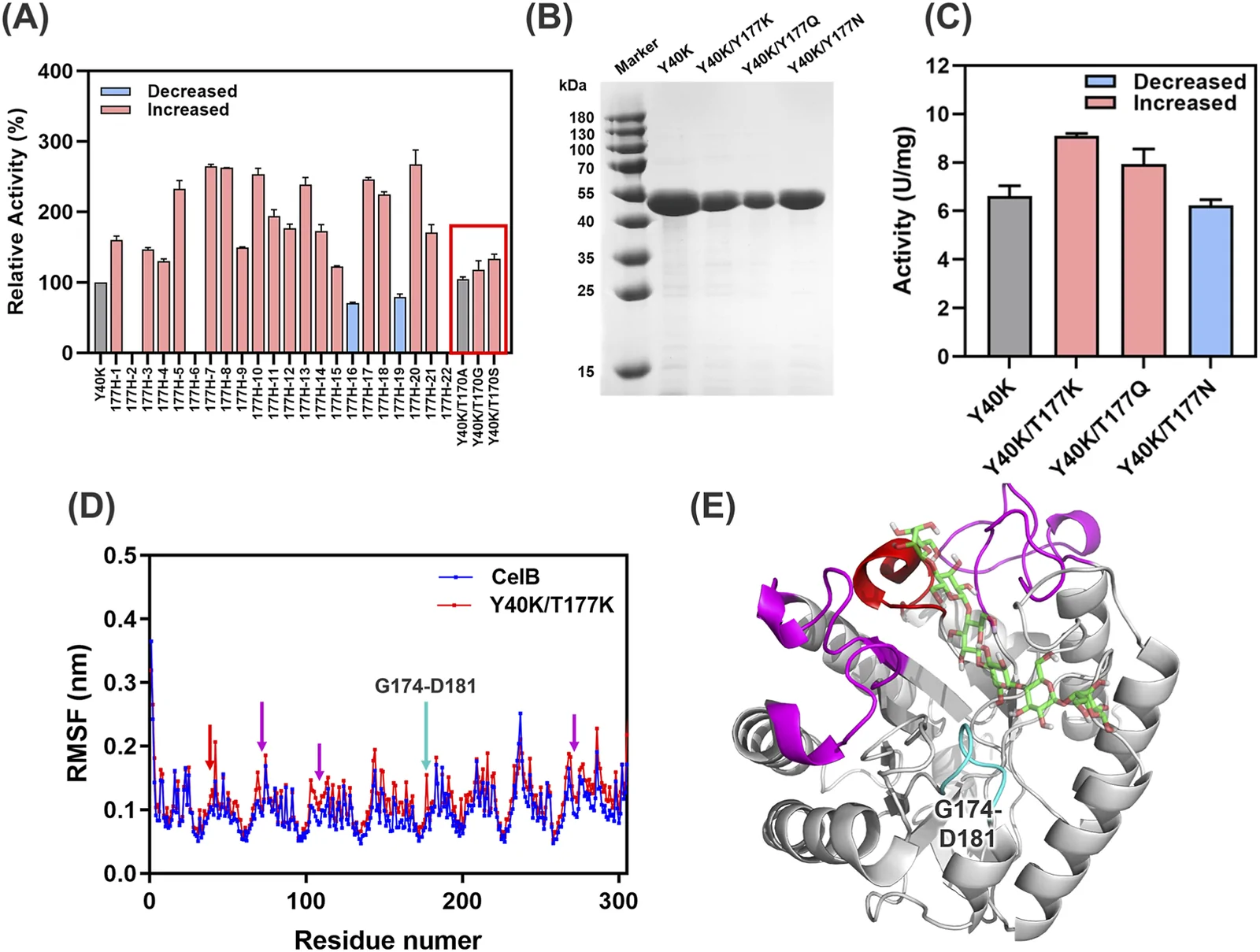
Iterative, focused saturated mutagenesis of Thr177 based on Y40K **(A)** Initial screening for focused library of Thr177. **(B)** Purification for the double mutants screened out from the library. **(C)** Specific activities of purified mutants towards CMC-Na (statistical significances were analyzed between the best Y40K/T177K and WT, ***p < 0.001) **(D)** RMSF comparison between Y40K/T177K and WT. **(E)** The introduction of T177K induced the flexibility enhancement of its local region (G174-D181, colored in cyan) based on Y40K.

### 3.5 Mutagenesis attempt on proline-riched linker

Additionally, we also noticed the distinctive PR-linker connecting the GH5-CD and ChtBD3 of CelB (Supplementary Figure S7A). A previous study on modular GH5 endoglucanases have shown that the length and flexibility of interdomain linkers can substantially influence catalytic behavior (Ruiz et al., 2016). Accordingly, four less-conserved proline residues (Pro313, Pro319, Pro333 and Pro334) were individually substituted with serine to examine whether modulating linker flexibility could further improve the catalytic performance of Y40K/T177K. All triple mutants were purified like other aforesaid variants (Supplementary Figure S7B). However, all triple mutants exhibited reduced activities relative to Y40K/T177K. Y40K/T177K/P319 S even showed 80% activity loss (Supplementary Figure S7C). These results indicate that the Pro-to-Ser substitutions within the PR-linker were detrimental to the catalytic performance of Y40K/T177K, suggesting that the native linker architecture may be important for maintaining an appropriate functional relationship between GH5-CD and ChtBD3. Thus, the linker substitutions examined here impaired rather than further improved catalytic performance.

### 3.6 Biochemical characterization

Biochemical characterization further confirmed the functional impact of site-directed mutagenesis. Temperature-activity profiles revealed pronounced differences in thermal adaptation among the variants (Figure 5A). The wild-type CelB exhibited an optimal reaction temperature of 65 °C. In contrast, progressive introduction of mutations led to a gradual reduction in optimal temperature: the single mutant Y40K displayed an optimum of 50 °C, while the double mutant Y40K/T177K exhibited a further reduced optimum of 45 °C. Furthermore, no detectable activity was observed for CelB at 20 °C. In contrast, Y40K and Y40K/T177K exhibited activities of 1.792 U/mg and 4.970 U/mg, indicating improved adaptation to lower temperatures. These results further suggest that the mutations effectively enhance the flexibility of the corresponding regions, thereby improving the adaption to medium-low temperatures. However, a trade-off also emerged at elevate temperatures (e.g., 70 °C and 80 °C), wherein the activities of the mutants were significantly lower than that of CelB. To further elucidate the structural basis underlying this trade-off, MD simulations were extended to 70 °C (343 K). RMSF analysis revealed that, compared with simulations at 300 K, both mutants exhibited substantial flexibility increases in the His35-Asn45 segment and the neighboring Ala64-Val78 region (Supplementary Figure S9A). This excessive flexibility may be attributed to an intricate intramolecular interaction network centered on His35-Asn45 (Supplementary Figure S9B). In addition to the previously reported hydrophobic packing between Tyr40 and His35/Lys267, Trp39 and Gln38 engage in T-shaped Pi-Pi stacking and Pi-donor hydrogen bonding with Tyr66, respectively. Consequently, such hyper-flexibility in the His35-Asn45 is likely to propagate to the neighboring Ala64-Val78, perturbing the structural integrity of the catalytic domain and ultimately leading to substantial activity loss at elevated temperatures.

**FIGURE 5:**
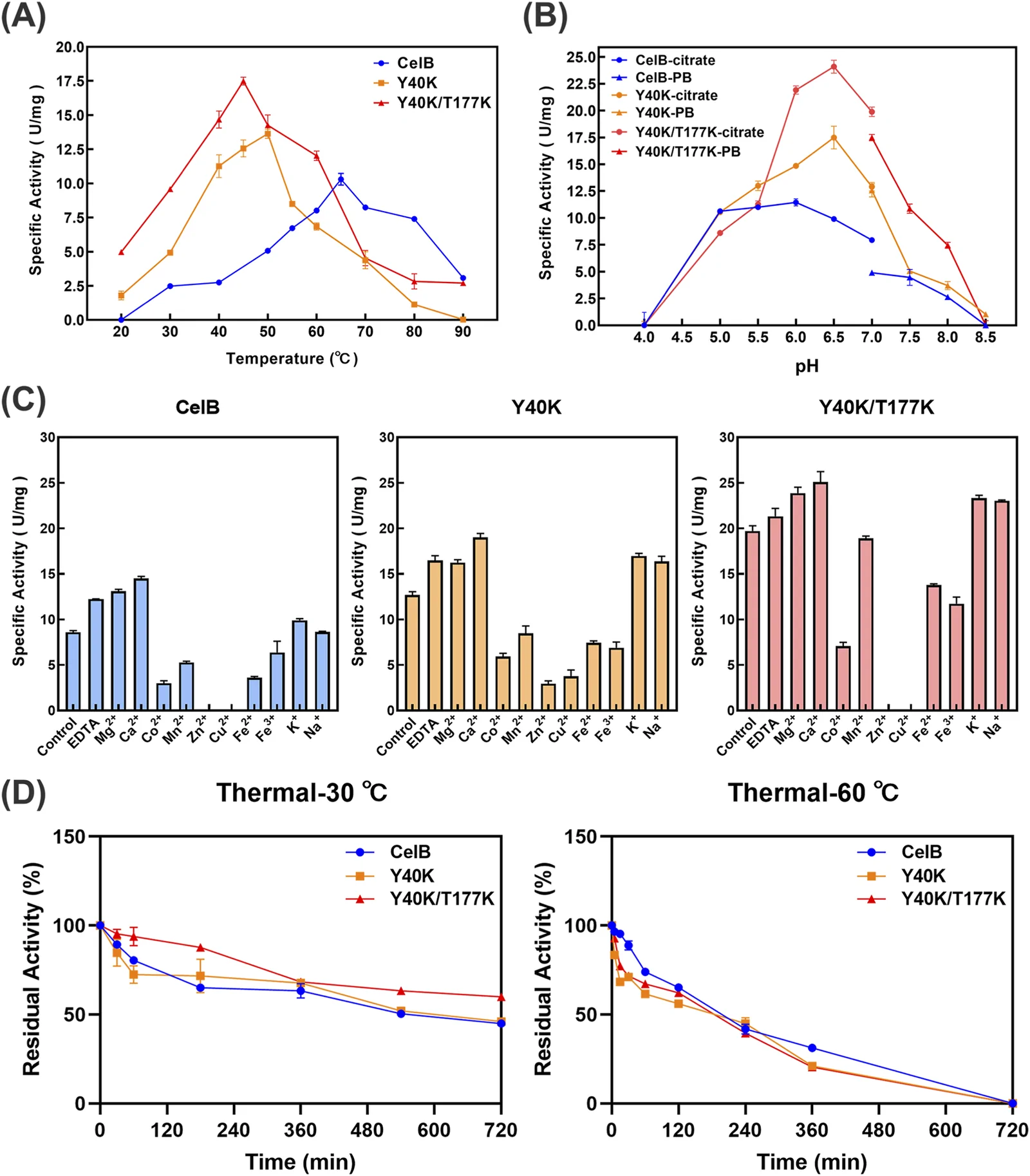
Biochemical characterization of CelB, Y40K and Y40K/T177K. **(A)** Temperature-activity profiles across 20 °C–90 °C. **(B)** pH-activity profiles spanning pH 4.0–8.5. Citrate-Na_2_HPO_4_ buffer (citrate) was used for the pH range 4.0–7.0, and phosphate buffer (PB) for pH 7.0–8.5. **(C)** Effect of metal ion treatment on enzymatic activity. **(D)** Thermal deactivation profiles under 30 °C and 60 °C over 0–12 h.

pH-activity profile (Figure 5B) revealed that both wild-type CelB and the two variants exhibited maximal activity under mildly acidic conditions (pH 6.0–6.5). In contrast, strongly acidic (pH 4.0) or alkaline (pH 8.5) conditions resulted in severe activity loss. The effects of 5 mM metal ions (Figure 5C) on wild-type CelB and two variants were similar. Mg^2+^, Ca^2+^ and K^+^ enhanced the activity of all three enzymes to varying extents, with K^+^ and Na^+^ exerting significantly stronger activation in Y40K and Y40K/T177K. In contrast, Co^2+^, Mn^2+^, Zn^2+^, Cu^2+^, Fe^2+^ and Fe^3+^ exerted inhibitory effects. Notably, Zn^2+^ and Cu^2+^ displayed the most potent inhibition, completely abolishing enzymatic activity in both wild-type CelB and the double mutant Y40K/T177K.

Thermal inactivation assays were conducted to evaluate the impact of mutagenesis on overall structural stability. As shown in Figure 5D, at 30 °C, CelB and its variants Y40K and Y40K/T177K exhibited comparable thermostability, with slow activity loss over time. After 12 h, all three enzymes retained >45% of their initial activity. To better resolve their differences, the incubation temperature was increased to 60 °C. Under this condition, their activities declined more rapidly. After 4 h, the remaining activities were approximately 40% for all three enzymes. After 6 h, the two variants retained ∼20% activity which was slightly below that of wild-type CelB (31%). Notably, all enzymes completely inactivated after 12-h incubation at 60 °C. DSF analyses further demonstrated that Y40K and Y40K/T177K exhibited prominently lower *T*_m_ values compared with CelB (Supplementary Figure S10). Specifically, the *T*_m_ of CelB was 65.2 °C. The incorporating of Y40K reduced the *T*_m_ towards 54.5 °C, and the further introduction of T177K led to a modest decrease to 52.4 °C. Combining with the results of temperature-activity profiles and the RMSF analyses under 343 K, the weakening of hydrophobic packing and the flexibility enhancement across multiple peripheral regions triggered by Y40K collectively diminish structural rigidity and compromise adaptability towards elevate temperatures.

The kinetic parameters of CelB, Y40K and Y40K/T177K were determined using CMC-Na and PASC as substrates (Table 2). The corresponding Michaelis-Menten plots for these substrates are presented in Supplementary Figure S11. For soluble CMC-Na, CelB exhibited a *K*_*m*_ of 4.371 mg/mL and a *k*_*cat*_ of 18.825 s^-1^. In contrast, when PASC was used as the substrate, CelB displayed reduced substrate affinity and catalytic efficiency, as evidenced by a higher *K*_*m*_ (5.867 mg/mL) and lower *k*_*cat*_ (8.944 s^-1^). This was attributed to the limited accessibility of hydrolysable glycosidic bonds within the insoluble PASC matrix, thereby restricting productive enzyme-substrate interactions (Petrášek and Nidetzky, 2025). The single mutant Y40K exhibited a nearly two-fold increase in *k*_*cat*_ (35.790 s^-1^) towards CMC-Na. However, a slight increase in *K*_*m*_ (4.930 mg/mL) was also observed. The introducing of T177K (Y40K/T177K) led to a further increase of *k*_*cat*_ (45.840 s^-1^) and lower *K*_*m*_ (3.896 mg/mL) than CelB. Similarly, for insoluble PASC, Y40K and Y40K/T177K showed progressive increases in *k*_*cat*_ (10.378 and 18.021 s^-1^ respectively). In contrast, neither of them showed a favorable effect on *K*_*m*_ for PASC. Taken together, Y40K/T177K achieved the highest catalytic efficiency, evidenced by *k*_*cat*_/*K*_*m*_ of 11.766 mL mg^-1^·s^-1^ towards CMC-Na and 2.719 mL mg^-1^·s^-1^ toward PASC, representing 2.73-fold and 1.78-fold increases over the wild-type enzyme.

**TABLE 2:** Kinetic constants of CelB, Y40K and Y40K/T177K towards CMC-Na and PASC. Values in parentheses denote the corresponding standard deviations.

Substrate	Enzymes	*K*_*m*_ (mg/mL)	*k*_*cat*_ (s^-1^)	*k*_*ca*t_/*K*_*m*_ (mL mg^-1^s^-1^)
CMC-Na	CelB	4.371 (0.434)	18.825 (1.289)	4.307
	Y40K	4.930 (0.369)	35.790 (1.681)	7.260
	Y40K/T177K	3.896 (0.290)	45.840 (2.181)	11.766
PASC	CelB	5.867 (0.345)	8.944 (0.357)	1.525
	Y40K	7.715 (0.905)	10.378 (1.076)	1.345
	Y40K/T177K	6.628 (0.221)	18.021 (0.489)	2.719

Regarding the effects on *K*_m_, in addition to the improvement of *k*_*cat*_, substrate accessibility and binding are also important determinants (Seibert and Tracy, 2021). Flexibility enhancements of peripheral regions may influence not only glycan access and release from the catalytic cleft but also the interactions involved in substrate binding. As shown in the non-bond interaction analysis (Figure 6A), the highly conserved His35 and Lys267 engage in hydrophobic packing with Tyr40 and collectively contribute to glycan binding. The imidazole sidechain of His35 forms a hydrogen bond (H-bond) with the sugar unit at −2 subsite between Nε2-H (donor) and C2-OH (acceptor). The amino group of Lys267 can also form H-bonds with the hydroxyl groups of sugar unit at −2 subsite (donor: ε-NH_3_^+^; acceptor: C2-OH). Weakening this hydrophobic packing as well as regional flexibility enhancement induced by Y40K will affect the orientation of His35 and Lys267, thus further affecting binding procedure. Similarly, in Figure 6B, the adjacent residues of Thr177 (Trp178 and Gln180) also participate in forming enzyme-substrate interactions. The pyrrole ring of Trp178 forms a weak Pi-sigma interaction with C3-H of sugar unit at subsite +1 while the amide amino group of Gln180 forms a H-bond with C3-OH of sugar unit at subsite +2. Hence, the flexibility enhancement of Gly174-Asp181 may also impact such interactions. These structural observations suggest that substitutions at Tyr40 and Thr177 may affect enzyme-glycan interactions in addition to altering peripheral conformational dynamics. Thus, the observed changes in *K*_*m*_ of Y40K and Y40K/T177K reflect the integrated influence of glycan accessibility, enzyme-substrate interactions and changes in catalytic turnover.

**FIGURE 6:**
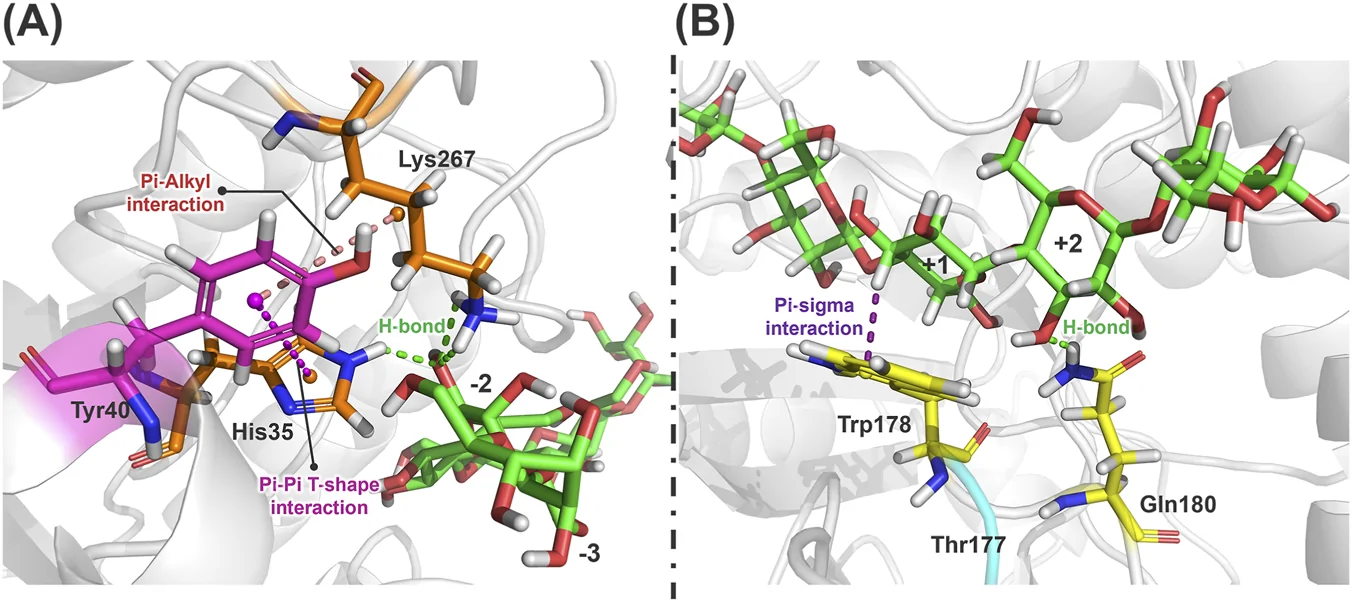
Enzyme-substrate interactions analysis around mutation site 40 **(A)** and 177 **(B)** using Discovery Studio 2021. For clarity in visualizing the local microenvironment, Thr177 was omitted to better resolve the spatial arrangement and interaction profiles of adjacent residues Trp178 and Gln180.

### 3.7 Effects of flexibility enhancement across multiple peripheral regions in Y40K/T177K

Previous work by Taylor et al. demonstrated that enhanced flexibility of the substrate-enclosing loops in *Tr*Cel7A correlates with increased ligand mobility and attenuated enzyme-substrate interactions, thereby facilitating enzyme dissociation from the cellulose chain upon encountering steric obstacles (Taylor et al., 2018). This earlier study provided a mechanistic precedent linking the dynamics of substrate-enclosing regions with enzyme-glycan separation, suggesting that strategic modulation of enzyme flexibility can be a powerful strategy to facilitate catalytic cycle of cellulases. We therefore investigated whether the augmented flexibility across multiple peripheral regions of the Y40K/T177K would create analogous functional consequences, thereby contributing to the observed increase in catalytic turnover.

Based on this premise, MD simulations were conducted on enzyme-cellohexaose complexes to elucidate the structural consequences induced by Y40K/T177K. As established in prior computational studies, SASA serves as a critical metric for assessing the openness of catalytic cavity and accessibility of functional sites (Stepankova et al., 2013; Haque and Prabhu, 2016; Distaso et al., 2023). As shown in Figure 7A, the catalytic cavity of Y40K/T177K possessed a higher SASA value of 5.49650 ± 0.86323 nm^2^ relative to that of CelB (4.08212 ± 0.57543 nm^2^). This notable increase reveals a more open and dynamic catalytic cavity in the Y40K/T177K, which reduces steric constraints, thereby promoting glycan chain mobility. To further substantiate this hypothesis, the dynamic changes of distance between the center of mass of the catalytic residues Glu139 and Glu228 (COM1) and that of the cellohexaose ligand (COM2) were monitored, as an indicator of the relative displacement of the bound glycan from the catalytic center. As illustrated in Figure 7B, the mean COM1-COM2 distance remained stable at 0.98765 ± 0.03285 nm in the CelB, whereas increased prominently to 1.24289 ± 0.08932 nm in the Y40K/T177K. This prominent increase indicated that the cellohexaose ligand preferentially sampled configurations farther removed from the catalytic center in the double mutant. Combination of SASA and COM-distance analyses suggest that the altered peripheral dynamics of Y40K/T177K lead to a more open catalytic cavity and reduced hydrogen-bonding interactions, which promote greater displacement of the bound glycan chain. This behavior is consistent with an increased tendency toward glycan dissociation from the catalytic cleft, thereby facilitating active-site re-availability for subsequent catalytic cycles and enhancing overall catalytic turnover. Such a mechanism parallels observations in endoglucanase *Tr*Cel5A, where a high dissociation rate from the cellulose chain correlates with improved turnover number (Kurašin and Väljamäe, 2011).

**FIGURE 7:**
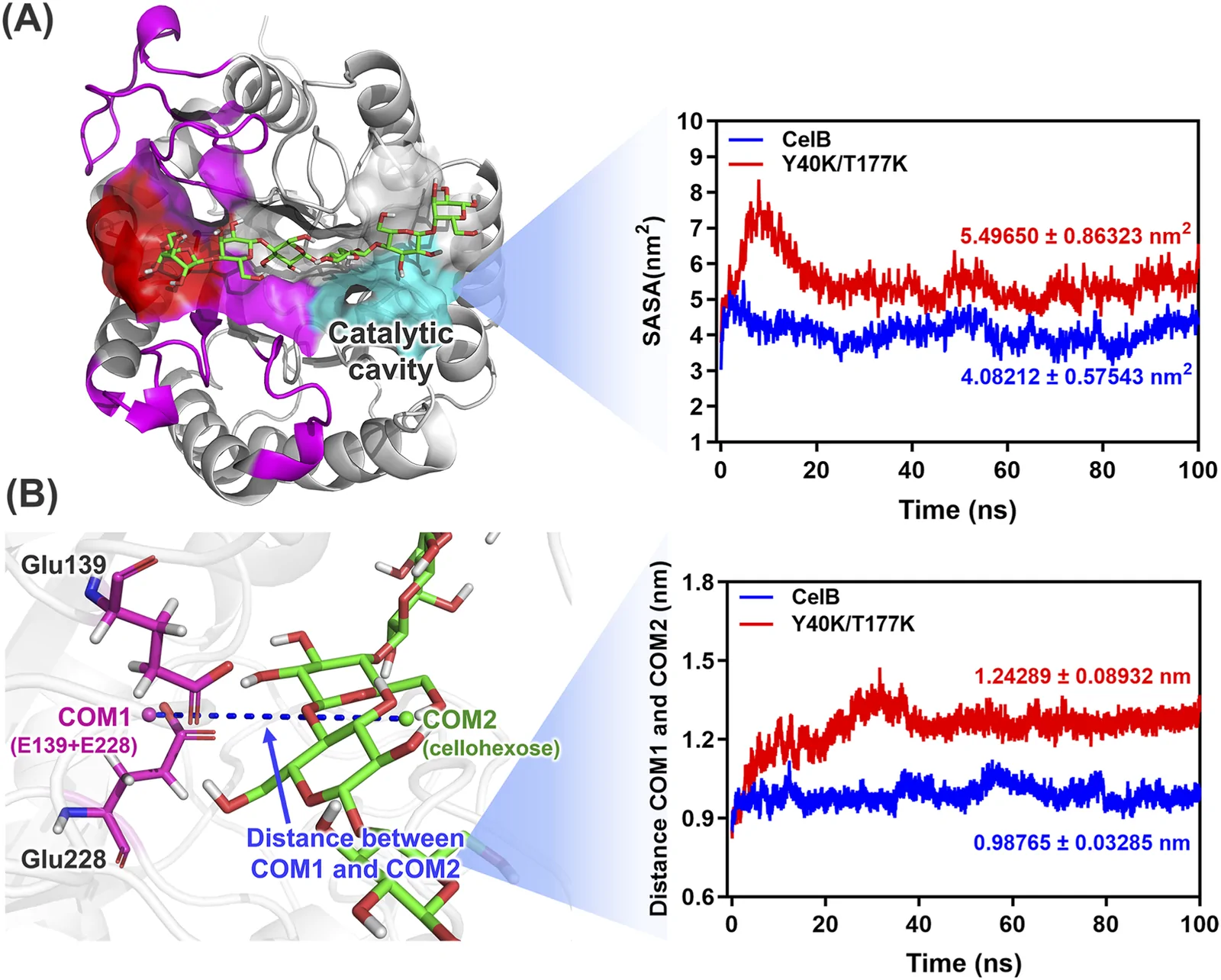
Structural insight into the effects induced by Y40K/T177K based on MD simulations. **(A)** SASA analysis of the catalytic cavity in CelB and Y40K/T177K. **(B)** The dynamic changes in the distance between the COMs of catalytic residues (Glu139, Glu228) and cellohexaose ligand in the enzyme-cellohexaose complexes of CelB and Y40K/T177K.

## 4 Discussion

Efficient and precise enzyme engineering represents a pivotal strategy for developing high-performance enzyme variants with promising industrial application prospects. A critical prerequisite for this process is the rational selection of engineering targets and appropriate mutation strategies. Herein, we focused on the peripheral regions of the GH5 family endoglucanase CelB and established a structure-guided engineering strategy.

In the first stage of structure- and sequence-guided mutagenesis, single mutants Y40G and T177 N were identified, exhibiting 1.64-fold and 1.55-fold increases in specific activity, respectively. These results initially suggest that enhancing the fluctuations of corresponding peripheral regions represent a promising strategy for improving specific activity. Compared with the structurally and functionally constrained interior of the catalytic cavity, such peripheral regions exhibit greater mutational tolerance, making them attractive targets for rational engineering. This rationale is corroborated by multiple reported beneficial mutations located in outer structural elements rather than the catalytic core, including T70Q (Lv et al., 2021) and K91I/A198 T (Lv et al., 2024) in, EG5C-1, the triple variant N193A/T300P/D307 P in *An*Cel5A (Zheng et al., 2022). Collectively, these findings support the potential of targeting non-conserved peripheral regions as an alternative to directly modifying the highly conserved catalytic center.

To further optimize the flexibility of peripheral regions, a tailored, focused mutagenesis library containing small or hydrophilic substituents bearing polar or charged sidechain was constructed. This strategy substantially reduced library complexity, alleviated the screening burden, and facilitated the identification of beneficial variants. Through iterative mutagenesis, Y40K/T177K was identified as the best-performing variant. Notably, Lys substitutions consistently produced the greatest improvements in activity at both position 40 and 177, highlighting their superior performance in this context. This observation is consistent with the findings of a prior mutagenesis study on the GH7 cellobiohydrolase I *Te*Cel7A (Zong et al., 2019). In this study, Thr393 (RSA = 50%, calculated based on PDB ID: 3 PFX), located on a peripheral, solvent-exposed loop (A4) flanking the product-binding site, was substituted with hydrophilic Lys (T393K). The substitution significantly increased regional flexibility and was associated with accelerated cellobiose release and catalytic cycling. The enhanced enzymatic activity conferred by the highly hydrophilic Lys substitution was attributed to changes in protein-solvent coupled dynamics associated with altered hydration. Likewise, in a modification study for potato protein, covalent conjugation of gallic acid enriched the population of surface-exposed hydrophilic patches. This alteration remodeled the interfacial hydration shell, leading to a more loosely packed, partially unfolded conformation characterized by increased backbone flexibility and enhanced solvent accessibility (Huang et al., 2026). Collectively, these findings suggest that introducing hydrophilic substitutions at selected solvent-exposed peripheral sites can provide a useful means of tuning local conformational dynamics, although their functional consequences remain site- and context-dependent.

Y40K/T177K showed prominently higher *k*_*cat*_/*K*_*m*_ towards CMC-Na and PASC, representing 2.73- and 1.78-fold enhancements over the wild-type CelB. The improved catalytic efficiency primarily resulted from the substantial increase in *k*_*cat*_. MD simulations provided structural insights into how Y40K/T177K-induced flexibility enhancement across multiple peripheral regions modulated catalytic performance. The results indicated that the altered peripheral dynamics of Y40K/T177K collectively promoted cavity opening and resulted in significantly greater displacement of cellohexaose from the catalytic center (Figures 6A,B). This behavior is consistent with an increased tendency toward glycan dissociation, thereby contributing to the observed increase in catalytic turnover (Zong et al., 2019). Nevertheless, these peripheral regions may also contribute directly to substrate recognition through specific enzyme-substrate interactions (Kumagai et al., 2015; Zheng et al., 2018). Consequently, excessive modification may impair functional integrity or compromise catalytic performance has also been reported in previous studies of GH5 enzymes.

In brief, high-performance GH5 endoglucanases are of considerable interest for industrial applications, especially in lignocellulosic biorefineries. This study advances the field by deliberately targeting non-conserved residues situated around solvent-exposed peripheral regions of a GH5 endoglucanase CelB, thereby broadening the rational framework for developing high-performance endoglucanase with enhanced industrial potential.

## Data Availability

The original contributions presented in the study are included in the article/Supplementary Material, further inquiries can be directed to the corresponding authors.
