# Supplementary material for: Enhancing the flexibility of the peripheral regions within the catalytic cavity of a family 5 glycoside hydrolase CelB for improving catalytic efficiency
Source: Front Bioeng Biotechnol. 2026 Oct 26;14:1931754. doi: 10.3389/fbioe.2026.1931754 (PMC13746197; doi:10.3389/fbioe.2026.1931754)
Supplement: Supplementary file 1 [file Table1.docx]

Supplementary Material

# 1 Supplementary Figures and Tables

## 1.1 Supplementary Tables

**Supplementary Table 1** Mutagenic primers for single-point mutations on CelB. The codons for substitutions were marked in bold fonts.

| **Primers** | **Sequences（5’-3’）** |
| --- | --- |
| W39F-1 | GACTTCAG**TTT**TACGGTCAGTTCGTTA |
| W39F-2 | TGACCGTA**AAA**CTGAAGTCCGTGAGAG |
| W39T-1 | GACTTCAG**ACT**TACGGTCAGTTCGTTA |
| W39T-2 | TGACCGTA**AGT**CTGAAGTCCGTGAGAG |
| Y40G-1 | TCAGTGG**GGT**GGTCAGTTCGTTAACTAC |
| Y40G-2 | AACTGACC**ACC**CCACTGAAGTCCGTGA |
| Y40I-1 | TCAGTGG**ATT**GGTCAGTTCGTTAACTAC |
| Y40I-2 | AACTGACC**AAT**CCACTGAAGTCCGTGA |
| T177N-1 | AACTGGA**AAT**TGGTCTCAGGATGTTCA |
| T177N-2 | TGAGACCA**ATT**TCCAGTTCCAACGATG |
| L210V-1 | GTCAAAAC**GTT**AGGGATCAAGTCGATT |
| L210V-2 | GATCCCTA**ACG**TTTTGACCGTGAGTAC |
| S265N-1 | GTCACTT**TCT**CACAAGGATGAGTCTA |
| S265N-2 | TCCTTGTG**AGA**AAGTGACCAGTTAGC |

**Supplementary Table 2** Primers for site-specific mutagenesis to introduce representative small residues for substitutions at Tyr40 and Thr177.

| **Primers** | **Sequences（5’-3’）** |
| --- | --- |
| Y40A-1 | TCAGTGG**GCC**GGTCAGTTCGTTAACTAC |
| Y40A-2 | AACTGACC**GGC**CCACTGAAGTCCGTGA |
| Y40V-1 | TCAGTGG**GTT**GGTCAGTTCGTTAACTAC |
| Y40V-2 | AACTGACC**AAC**CCACTGAAGTCCGTGA |
| Y40S-1 | TCAGTGG**AGT**GGTCAGTTCGTTAACTAC |
| Y40S-2 | AACTGACC**ACT**CCACTGAAGTCCGTGA |
| T177G-1 | AACTGGA**GGT**TGGTCTCAGGATGTTCA |
| T177G-2 | TGAGACCA**ACC**TCCAGTTCCAACGATG |
| T177A-1 | AACTGGA**GCT**TGGTCTCAGGATGTTCA |
| T177A-2 | TGAGACCA**AGC**TCCAGTTCCAACGATG |
| T177S-1 | AACTGGA**TCT**TGGTCTCAGGATGTTCA |
| T177S-2 | TGAGACCA**AGA**TCCAGTTCCAACGATG |

**Supplementary Table 3** Degenerate primers for iterative, focused saturated mutagenesis of Tyr40 and Thr177 to introduced hydrophilic residues.

| **Primers** | **Sequences（5’-3’）** |
| --- | --- |
| Y40 (H)-1 | TCAGTGG**VAW**GGTCAGTTCGTTAACTAC |
| Y40 (H)-2 | AACTGACC**WTB**CCACTGAAGTCCGTGA |
| T177 (H)-1 | AACTGGA**VAW**TGGTCTCAGGATGTTCA |
| T177 (H)-2 | TGAGACCA**WTB**TCCAGTTCCAACGATG |

**Supplementary Table 4** Residue types included in the hydrophilic library encoded by degenerate codon “^5’^-VAW-^3’^”.

| **^5’^-VAW-^3’^** | **Residues** |
| --- | --- |
| AAA | Lys |
| AAT | Asn |
| CAA | Gln |
| CAT | His |
| GAA | Glu |
| GAT | Asp |

## 1.2 Supplementary Figures

**Supplementary Figure 1.** Identification of bi-modular CelB. (A) Phylogenetic analysis based on a bi-modular GH5 endoglucanase *Bs*Egls (UniProt ID: A5A671, 499 aa) isolated from *Bacillus subtilis* 168. (B) Structure view of enzyme candidates selected from phylogenetic tree. (C) Expression detection of three enzymes candidates (BL0147, CelN and CelB).

**Supplementary Figure 2.** Conservation scoring of CelB generated by Consurf server.

**Supplementary Figure 3.** Relative solvent-accessible surface area (RSA) and Consurf-derived evolutionary profiling of Trp39, Tyr40, Thr177, Leu210, Thr265. Residues selected for substitution were highlighted in red.

**Supplementary Figure 4.** Expression detection of W39T compared to W39F at same site.

**Supplementary Figure 5.** Whole plasmid mutagenetic PCR to construct focused libraries at site Tyr40 (A) and Thr177 (B).

**Supplementary Figure 6.** Expression detection of several mutants selected from the focused libraries of Thr177.

**Supplementary Figure 7.** Further superimposition of mutations on PR-linker to construct triple mutants. (A) Structural view of PR-linker. (B) Purification result of triple mutants. (C) Activity detection of triple mutants.

**Supplementary Figure 8.** Summary of RMSD curves for CelB (A), Y40G (B), T177N (C), Y40K (D), and Y40K/T177K (E). MD simulations for each enzyme were performed in triplet.

**Supplementary Figure 9.** Effects of the Y40K and Y40K/T177K mutations on Conformational Flexibility of CelB at 70 °C. (A) RMSF profiles of wild-type CelB, the Y40K single mutant, and the Y40K/T177K double mutant. Regions exhibiting significantly elevated RMSF values were highlighted. (B) Intramolecular interaction network centered on His35-Asn45 and its neighboring regions.

**Supplementary Figure 10.** *T*_m_ determination for CelB, Y40K and Y40K/T177K using differential scanning fluorimetry (DSF), with *T*_m_ values extracted from the peaks of the first derivative (dF/dT) of the fluorescence melting curves.

**Supplementary Figure 11.** Michaelis-Menten plots depicting the steady-state kinetic behavior of CelB and its variants Y40K and Y40K/T177K toward CMC-Na and PASC.
